# 3-D Visualization and Quantitation of Microvessels in Transparent Human Colorectal Carcinoma

**DOI:** 10.1371/journal.pone.0081857

**Published:** 2013-11-29

**Authors:** Yuan-An Liu, Shien-Tung Pan, Yung-Chi Hou, Ming-Yin Shen, Shih-Jung Peng, Shiue-Cheng Tang, Yuan-Chiang Chung

**Affiliations:** 1 Department of Chemical Engineering, National Tsing Hua University, Hsinchu, Taiwan; 2 Department of Pathology, Miaoli General Hospital, Miaoli, Taiwan; 3 Department of Surgery, National Taiwan University Hospital - Hsinchu, Branch, Hsinchu, Taiwan; 4 Institute of Biotechnology, National Tsing Hua University, Hsinchu, Taiwan; 5 Department of Medical Science, National Tsing Hua University, Hsinchu, Taiwan; 6 Department of Surgery, Cheng Ching General Hospital, Chung Kang Branch, Taichung, Taiwan; National Cancer Institute, National Institutes of Health, United States of America

## Abstract

Microscopic analysis of tumor vasculature plays an important role in understanding the progression and malignancy of colorectal carcinoma. However, due to the geometry of blood vessels and their connections, standard microtome-based histology is limited in providing the spatial information of the vascular network with a 3-dimensional (3-D) continuum. To facilitate 3-D tissue analysis, we prepared transparent human colorectal biopsies by optical clearing for in-depth confocal microscopy with CD34 immunohistochemistry. Full-depth colons were obtained from colectomies performed for colorectal carcinoma. Specimens were prepared away from (control) and at the tumor site. Taking advantage of the transparent specimens, we acquired anatomic information up to 200 μm in depth for qualitative and quantitative analyses of the vasculature. Examples are given to illustrate: (1) the association between the tumor microstructure and vasculature in space, including the perivascular cuffs of tumor outgrowth, and (2) the difference between the 2-D and 3-D quantitation of microvessels. We also demonstrate that the optically cleared mucosa can be retrieved after 3-D microscopy to perform the standard microtome-based histology (H&E staining and immunohistochemistry) for systematic integration of the two tissue imaging methods. Overall, we established a new tumor histological approach to integrate 3-D imaging, illustration, and quantitation of human colonic microvessels in normal and cancerous specimens. This approach has significant promise to work with the standard histology to better characterize the tumor microenvironment in colorectal carcinoma.

## Introduction

Formation of new blood vessels, or angiogenesis, helps tissue generation and regeneration in conditions such as embryonic growth and after injury [1,2]. In cancer development, the concept of tumor growth driven by angiogenesis is well accepted, which emphasizes the roles of new blood vessels in providing tumor cells with oxygen and nutrients for proliferation and in inducing distant metastasis by allowing the tumor cell to enter the circulation from immature neovessels [3,4]. To employ this concept in tumor analysis, Weidner et al. developed the index of microvessel density (MVD) by counting the number of tumor microvessels on histological slides to evaluate the angiogenic activity [5,6]. Since the 1990s, MVD has been demonstrated to be a valuable prognostic indicator in various types of malignancies [7-10], including colorectal carcinoma [11-13]. 

Although MVD and other microtome-based tissue analyses, such as hematoxylin and eosin (H&E) staining, are the gold standard in assessment of malignancy, the 2-dimensional (2-D) histological data are intrinsically challenged in examining the spatial features of the tumor architecture. For example, the opaque biopsy of colorectal carcinoma demands a thin tissue section, typically less than 5 μm, for the standard histological examination. Although experienced investigators can apply serial sectioning microscopy to generate consecutive tumor images [14,15] or conceive a virtual 3-dimensional (3-D) vascular structure based on the 2-D micrographs, the artifacts and/or the disconnected information caused by microtome slicing create difficulties to reconstruct the tissue network with precision. Due to the technical difficulties, information about the 3-D morphologies of the tissue networks, including those of the blood vessels, lymphatic vessels, and nerves, are generally not available in the literature to help understand their remodeling and the morphological patterns in lesion progression and cancer development.

To develop a microtome-free 3-D imaging method, we previously established a penetrative microscopic approach based on preparation of transparent tissues [16-20] (or “optical clearing” [21-24]: use of immersion solution to reduce scattering as light travels in the specimen) for 3-D imaging and illustration of mouse gut [25-29] and human enteric nervous system [18,19,30]. Here, in imaging of the human colorectal carcinoma, we applied the same optical approach with CD34 immunohistochemistry to acquire the spatial information of microvessels with high definition. Taking advantage of the voxel-based image data, we reconstructed the tumor microstructure and vasculature with a 3-D space continuum to perform analysis of tissue morphology in a global and integrated fashion. Examples of the 3-D features were given to illustrate the association between the tumor outgrowth and vasculature and to reveal the difference between the 2-D and 3-D quantitation of microvessels. The development of this vascular imaging approach and its application for qualitative and quantitative analyses of blood vessels in colorectal carcinoma are presented and discussed in this report.

## Materials and Methods

### Human specimens

Collection and use of human tissues were approved by the Institutional Review Board of National Taiwan University Hospital - Hsinchu Branch with written consent from the patients to use the obtained tissues. Colonic tissues were derived from colectomies carried out for patients with adenocarcinoma. The removed tissues were first perfused with phosphate-buffered saline (PBS) through the mesenteric artery to flush the residual blood. Afterward, tissues were fixed with 4% paraformaldehyde solution for 30 minutes at room temperature and then post-fixed in 4% paraformaldehyde solution for two hours at 4°C before being transferred to 0.1% paraformaldehyde for preservation. Specimens were later sectioned to ~300 µm in thickness by vibratome before being applied for tissue labeling. Normal areas of the colonic tissue were defined as and collected from locations at least 5 cm away from the tumor site. Overall, 27 image stacks derived from two patients were used to generate representative information. Table 1 lists the gender, age, and location of the sampled colon segments of the two subjects.

**Table 1:** Spatial microvessel density of normal colon mucosa^a^.

	Gender/Age (year)	Segment	Spatial microvessel density^b^ (microvessel volume/tissue volume) × 100%
Subject 1	Male/80	Sigmoid colon	2.1 ± 0.45
Subject 2	Female/59	Transverse colon	2.5 ± 0.46

^a^ Mucosal specimens were acquired from locations at least 5 cm away from the tumor site.

^b^ Eight 200-μm image stacks, with 81 consecutive optical sections in each image stack, were used to quantify the spatial microvessel density of each subject. Data are presented as means ± SD. The average density derived from the two subjects is 2.3% with no statistical difference (*P* = 0.18).

### Tissue labeling

The fixed specimens were immersed in 2% Triton X-100 solution for two hours at 15°C for permeabilization. The primary antibody used to reveal the vascular endothelium was a monoclonal mouse anti-CD34 antibody (Bio SB, Santa Barbara, CA, USA, cat# BSB 5230). Before applying the antibody, the tissue was rinsed in PBS. Afterward, the tissue was immersed in blocking buffer (2% Triton X-100, 10% normal goat serum, and 0.02% sodium azide in PBS). The primary antibody was then diluted in the dilution buffer (1:100, 0.25% Triton X-100, 1% normal goat serum, and 0.02% sodium azide in PBS) to replace the blocking buffer and incubated overnight at 15°C. Examination of the immunostaining variables is presented in Figures S1 and S2.

An Alexa Fluor 633-conjugated goat anti-mouse secondary antibody (1:200, Invitrogen, Carlsbad, CA, USA) was used to reveal the CD34-labeled vascular epithelium. Afterward, nuclear staining by propidium iodide (50 μg/ml, Invitrogen) was performed at room temperature for one hour. The labeled specimens were then immersed in the optical-clearing solution FocusClear^TM^ (CelExplorer, Hsinchu, Taiwan) overnight before confocal microscopy [31].

To integrate the standard histology with 3-D microscopy, we performed H&E staining and immunohistochemistry after 3-D microscopy: first, the tissue block was extensively washed with PBS for ten minutes to remove the optical-clearing reagent; afterward, embedding and microtome sectioning were performed to generate 4-μm tissue slices for H&E staining by Leica Autostainer XL (Leica, Deerfield, Illinois, USA) and immunohistochemistry by the automated staining machine (Ventana Medical Systems, Oro Valley, AZ, USA) with the ultraView Universal DAB Detection Kit (Ventana, with horseradish peroxidase conjugated secondary antibody against mouse IgG) to reveal the immunolabeled microvessels. The slide was counterstained with hematoxylin in immunohistochemistry.

### Confocal microscopy

The Zeiss LSM 510 Meta confocal microscope equipped with the objectives of 5× and 10× “Fluar” lenses was used to acquire the gross images of the colonic specimens. The 25× LD “Plan-Apochromat” glycerine immersion lenses (working distance: 570 µm) were used to acquire the high-resolution images (optical section: 5 µm; Z-axis increment: 2.5 µm). Each confocal micrograph consisted of 1,024 (X) × 1,024 (Y) pixels. The laser-scanning process was operated under the multi-track scanning mode to sequentially acquire signals in multiple channels, including the transmitted light channel. The Alexa Fluor 633-labeled vasculature was excited with a 633-nm laser source and the fluorescence was collected by the 650- to 710-nm band-pass filter. The propidium iodide-labeled nuclei were excited with a 543-nm laser source and the signals were collected by the 560- to 615-nm band-pass filter. 

### Image processing, projection, and analysis

The Avizo 6.2 image reconstruction software (VSG, Burlington, MA, USA; previously known as Amira), the Zen software (Carl Zeiss, Jena, Germany), and the LSM 510 software (Carl Zeiss) were used for analysis, processing, and projection of the confocal image stacks. The Avizo software was operated under a Dell T7500 workstation with a Linux operating system. Azivo’s “Noise Reduction Median” algorithms were used for background noise reduction. Feature extraction and image segmentation were performed by the “Label Field” function of Avizo to collect the voxels of vasculature for projection and analysis of the microvessel density. In Videos S1 and S2, the image stacks were displayed using the “Ortho Slice” function. The videos were made using the “Movie Maker” function with the increase in display time in association with the depth of the optical section. The “Voltex” module was used to project the 3-D images. The “Camera Rotation” function was used to create the 360-degree panoramic displays of the 3-D images.

Quantitation of microvessels was performed to analyze both the normal and adenocarcinoma specimens. An image stack with a tissue volume of 460 (X) μm × 460 (Y) μm × 200 (Z) μm ≈ 42 ×10^6^ μm^3^ (or 0.042 mm^3^) was used to acquire the spatial microvessel density (microvessel volume/tissue volume). In each image stack, 81 consecutive optical sections were used in quantitation. The CD34^+^ voxels and their spatial connections were used to define the enclosed regions to calculate the space occupied by the microvessels. The “Label Field” and “Material Statistics” functions of Avizo were applied to mark and count the voxels of microvessels. The spatial microvessel density was defined as the percentage of the voxels occupied by the microvessels over the overall tissue voxels. 

In 2-D microvessel analysis, we standardized the test by examining the microvessels at focal depths of 15, 30, and 60 μm under the tissue surface of a 200-μm image stack to avoid the disturbances from sample preparation and microscopy. One microvessel was defined as an area enclosed by the CD34 signals with distinct lumen. Quantitation was performed by three independent examiners, including one experienced pathologist (S.T.P.). Microvessels were counted on a 460×460-µm^2^ (0.21 mm^2^) micrograph and then averaged over the three inspections. Both the 2-D and 3-D tissue analyses were performed on the same image stacks.

### Statistics

The quantitative values are presented as means ± standard deviation (SD). Statistical differences were determined by the unpaired Student’s *t* test. Differences between groups were considered statistically significant when *p* < 0.05.

## Results

### Optical clearing of human colonic mucosa and colorectal carcinoma

**Figure 1:**
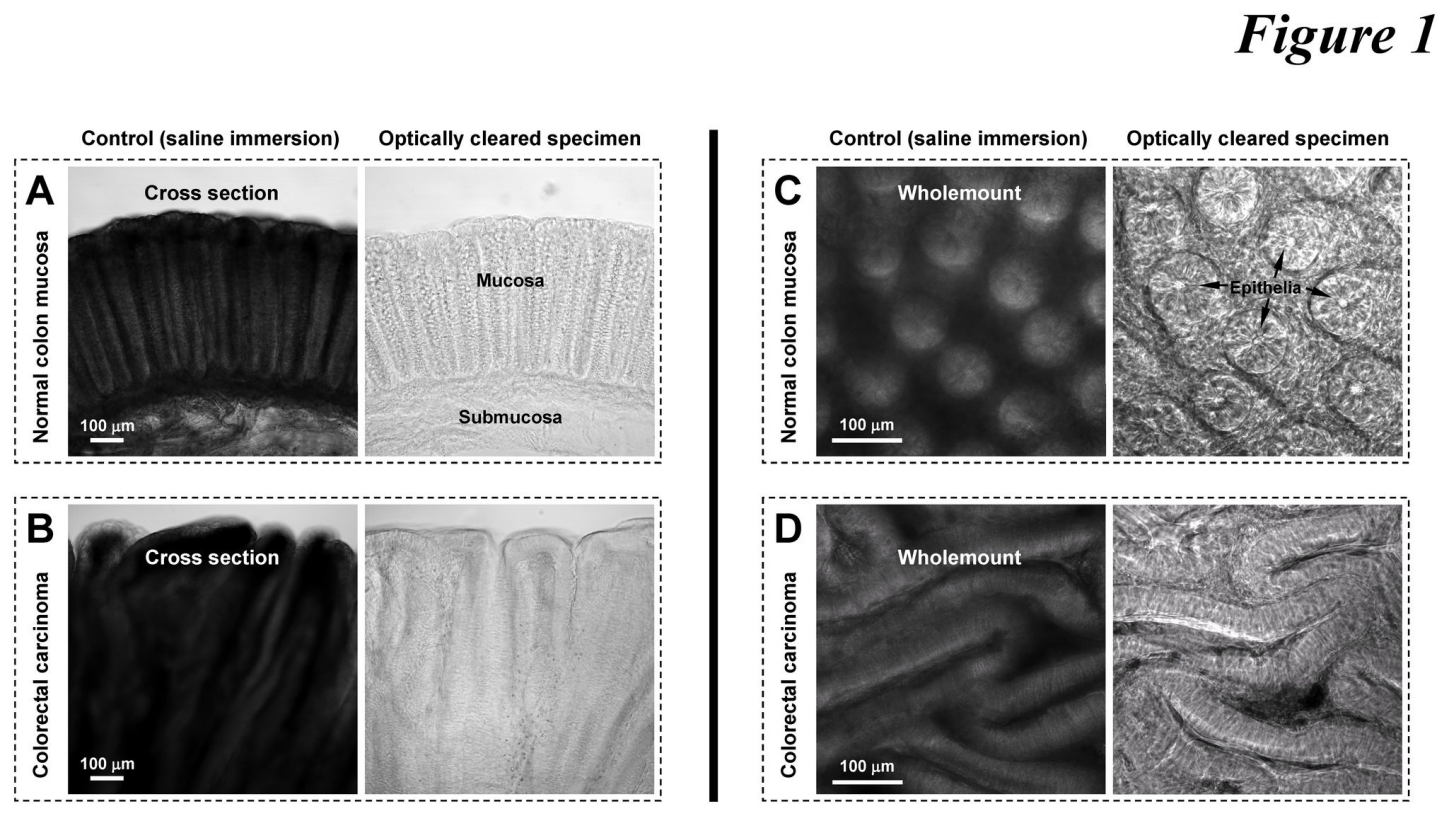
Optical clearing of human colorectal biopsies. (**A** and **B**) Cross sections of normal mucosa and colorectal carcinoma. (**C** and **D**) Wholemounts of normal mucosa and colorectal carcinoma. Arrows in panel C indicate the honeycomb-like crypt structure. In panel D, the honeycomb-like crypt epithelia changed to extended layers of epithelia in colorectal carcinoma. Both normal and diseased colons were sectioned by vibratome to specimens of 300 μm in thickness prior to saline or clearing immersion.

Both the normal human colonic mucosa and tissues of colorectal carcinoma are intrinsically opaque in saline (Figure 1, left panels). The opacity is due to the strong light scattering in the tissue which limits photon penetration. We reduced the scattering by immersing colonic specimens in the optical-clearing solution of high refractive index, similar to that of the tissue constituents (~1.46) [32,33], to promote photon penetration. Significantly, the improved tissue transparency allowed us to directly identify the colonic structures by transmitted light microscopy (Figure 1, right panels). For example, Figures 1A and C show the optically cleared crypt epithelium ascending from the crypt base toward the lumen and the honeycomb-like pattern of crypts in the normal mucosa, respectively. In transparent tumor biopsies, we observed the deformed colorectal epithelia in layers changing from the organized crypts in the mucosa (Figures 1B and D), which cannot be clearly seen in the opaque specimen.

### Optical clearing enables deep-tissue vascular microscopy

**Figure 2:**
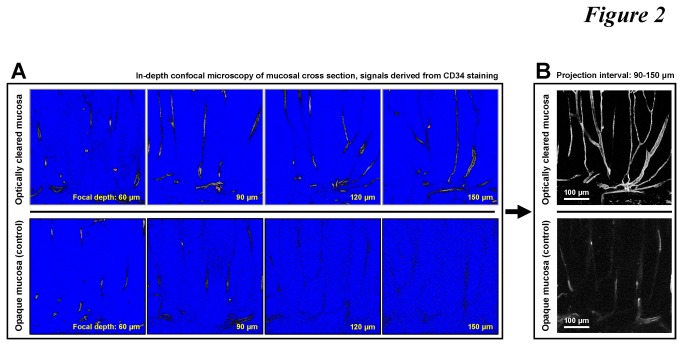
Deep-tissue microscopy of microvessels in normal colon mucosa. (**A**) Extended imaging depth in confocal microscopy with optical clearing. Upper panels show the CD34 signals of mucosal microvessels in the optically cleared mucosa, while in the opaque mucosa (lower panels, saline immersion) the signals drastically declined as the focal plane progressed into the tissue. Signal intensities are presented in grayscale, red (signal saturation), and blue (no signals). Video S1 compares the image stacks derived from the two optical conditions as the focal depth progressed from 0 to 150 μm. (**B**) Projection of the vascular signals derived from the optically cleared and opaque colon mucosa.

To label the vasculature, we examined the tissue staining conditions (including the source of the antibody and incubation time) (Figures S1 and S2) to reveal the colonic vascular network by CD34 immunostaining of the vascular endothelium. Next, we characterized the increase in the imaging depth of the CD34-labeled vasculature in the optically cleared tissue, relative to that in the untreated saline-immersion control (Figure 2). As can be seen, because of scattering, a drastic decline of the fluorescence signals along the focal depth occurred in the opaque control in comparison with the signals from the optically cleared tissue, particularly in the 90-150 μm interval (Figure 2A and Video S1). At 150 μm, loss of signals from the untreated tissue made feature recognition unfeasible. On the other hand, at the same imaging depths, the confocal micrographs derived from the optically cleared specimen maintained the signal contrast, leading to projection of the colonic vasculature with high definition (Figure 2B).

### Panoramic visualization of colonic microstructure and vasculature

**Figure 3:**
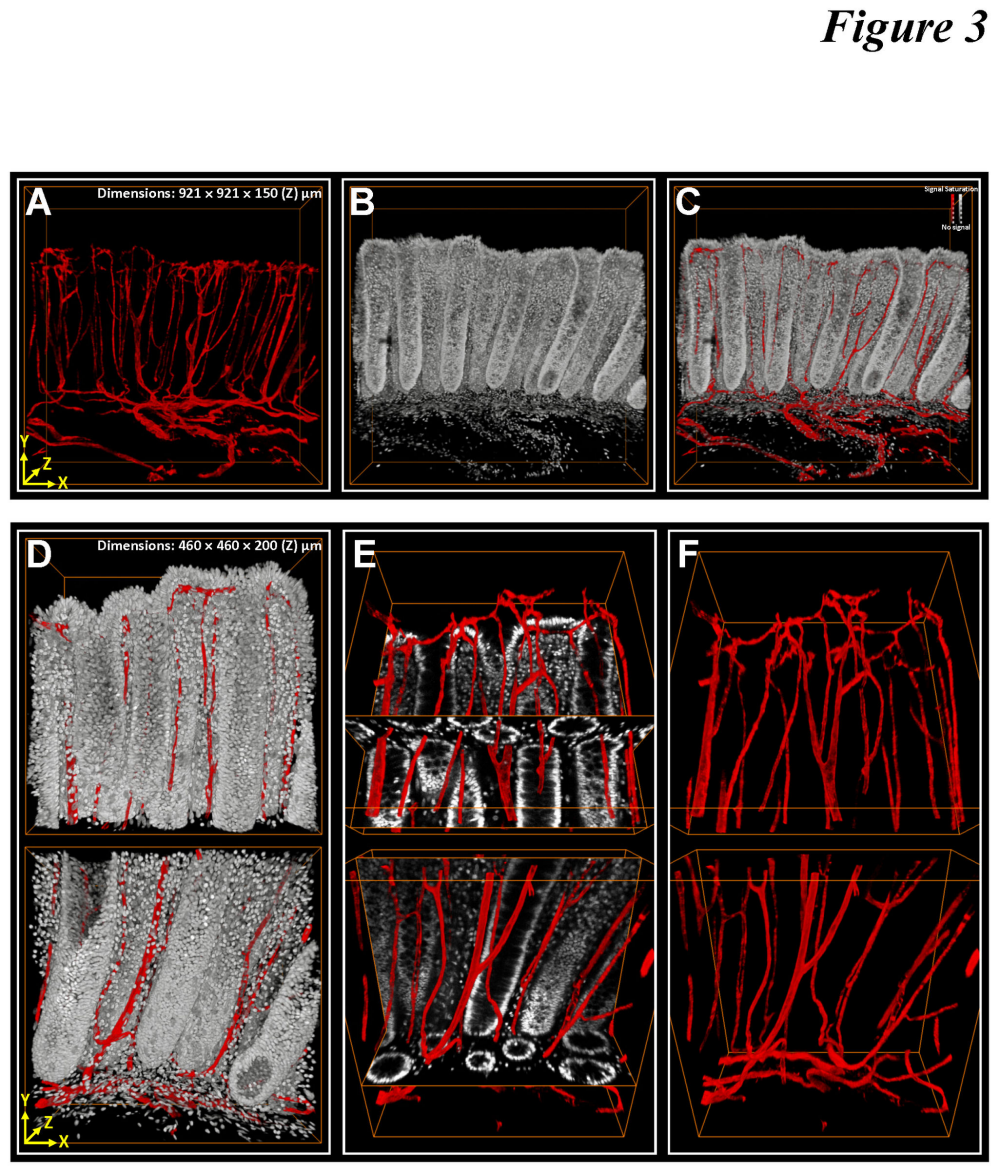
3-D image rendering and projection of colonic microstructure and vasculature. (**A**-**C**) Gross views of individual and merged projections of colonic crypts (gray: nuclei) and their surrounding vasculature (red: CD34). (**D**-**F**) Zoom-in examination of upper and lower parts of the crypts. In panel E, merged projection of vasculature with the orthogonal view of the crypt structure allows the interior domain of the scanned volume to be examined.

Figures 3A-C and Video S2 show a typical confocal image stack derived from the transverse section of the optically cleared colonic mucosa away from the tumor site. In the video, the serial optical sections along the focal path provide the volumetric information to depict the colonic microstructure and vasculature for in-depth projection (Figures 3A-C).

When zoomed in, we targeted both the top and lower parts of the mucosa to visualize the scanned volume with high definition. Figures 3D-F show the in-depth projection of the colonic crypts and vasculature. Note that unlike the tissue information derived from the microtome sections, the continuous anatomic information shown in Figure 3 and Video S2 examines a region of the mucosa. In the projection, we show that the peri-cryptic capillaries are embedded in the lamina propria, ascending along the crypt axis toward the openings. The example demonstrates that the spatial information of the mucosal components can be acquired and resolved in penetrative 3-D microscopy, which otherwise cannot be easily portrayed by the 2-D micrographs.

### Matched tissue information in 3-D and 2-D histology

Because the process of optical clearing is reversible with saline washing [34-37] and the procedure of confocal imaging is noninvasive to the specimen, we next investigated the feasibility of performing 3-D histology prior to the standard H&E staining and immunohistochemistry for potential integration of the two analytical approaches. 

**Figure 4:**
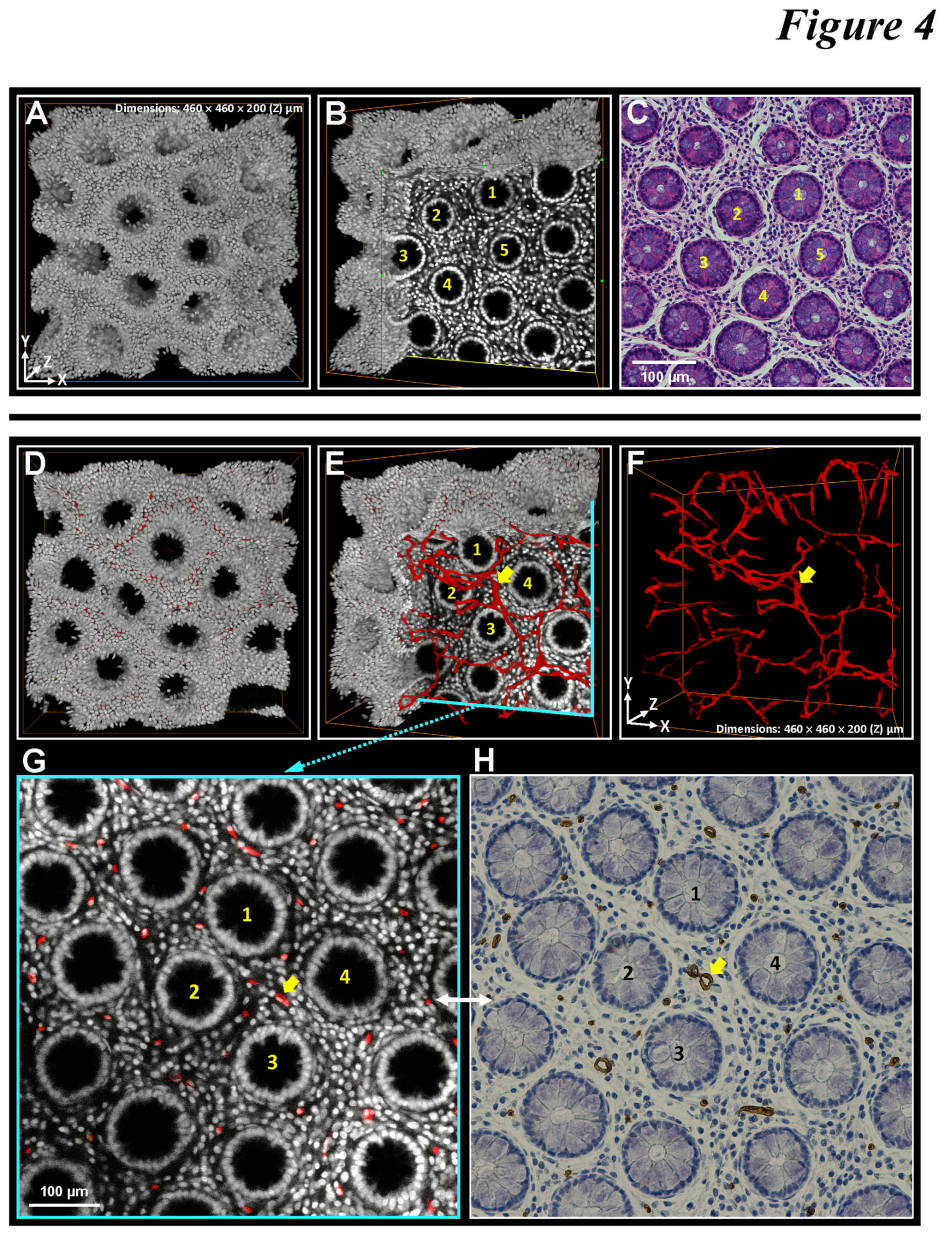
Matched tissue information derived from 3-D and 2-D histology. (**A**-**C**) Matched crypt morphology in micrographs derived from 3-D microscopy and H&E staining. Panels A and B: 3-D projections of the nuclear signals and one of the images derived from the confocal image stack. Panel C: H&E image of the same specimen used in (A) and (B). The yellow numbers in (B) and (C) indicate the same crypts found in the 3-D and 2-D images. (**D**-**F**) 3-D projection of the mucosal microstructure and vasculature. Panel D: merged projection of the nuclear (gray) and CD34 (red) signals. In panel E, the nuclear signals were digitally removed at a corner to reveal the embedded vasculature. Panel F: projection of the CD34 signals. (**G** and **H**) Counterparts of mucosal microstructure and vasculature revealed in 3-D and 2-D histology. Panel G: enlarged micrograph derived from the confocal image stack shown in panel E. Panel H: image derived from the standard CD34 immunostaining of the same specimen used in panels D-F (the specimen was retrieved after 3-D microscopy to perform standard histology). The numbers and arrows in panels E-H indicate the same crypts and microvessel.

Figure 4 demonstrates the matched structural information of colonic mucosa after performing the 3-D and 2-D histology. In this test, we first conducted 3-D microscopy with nuclear and CD34 staining (Figures 4A-B and D-G and Video S3) and then washed the specimens with saline to remove the clearing reagent. Afterward, the specimens were processed with standard histological procedure for H&E staining and CD34 immunohistochemistry to perform 2-D tissue analysis (Figures 4C and H). Using crypts as the landmark, we matched the H&E micrograph with one of the confocal micrographs acquired from the 3-D imaging (Figures 4A-C). Also, the counterparts of the CD34-labeled microvessels can be found in the 3-D and 2-D images (Figures 4D-H). These results confirm the compatibility of the two histological approaches for their integration.

### 3-D imaging and illustration of microvasculature in adenocarcinoma

**Figure 5:**
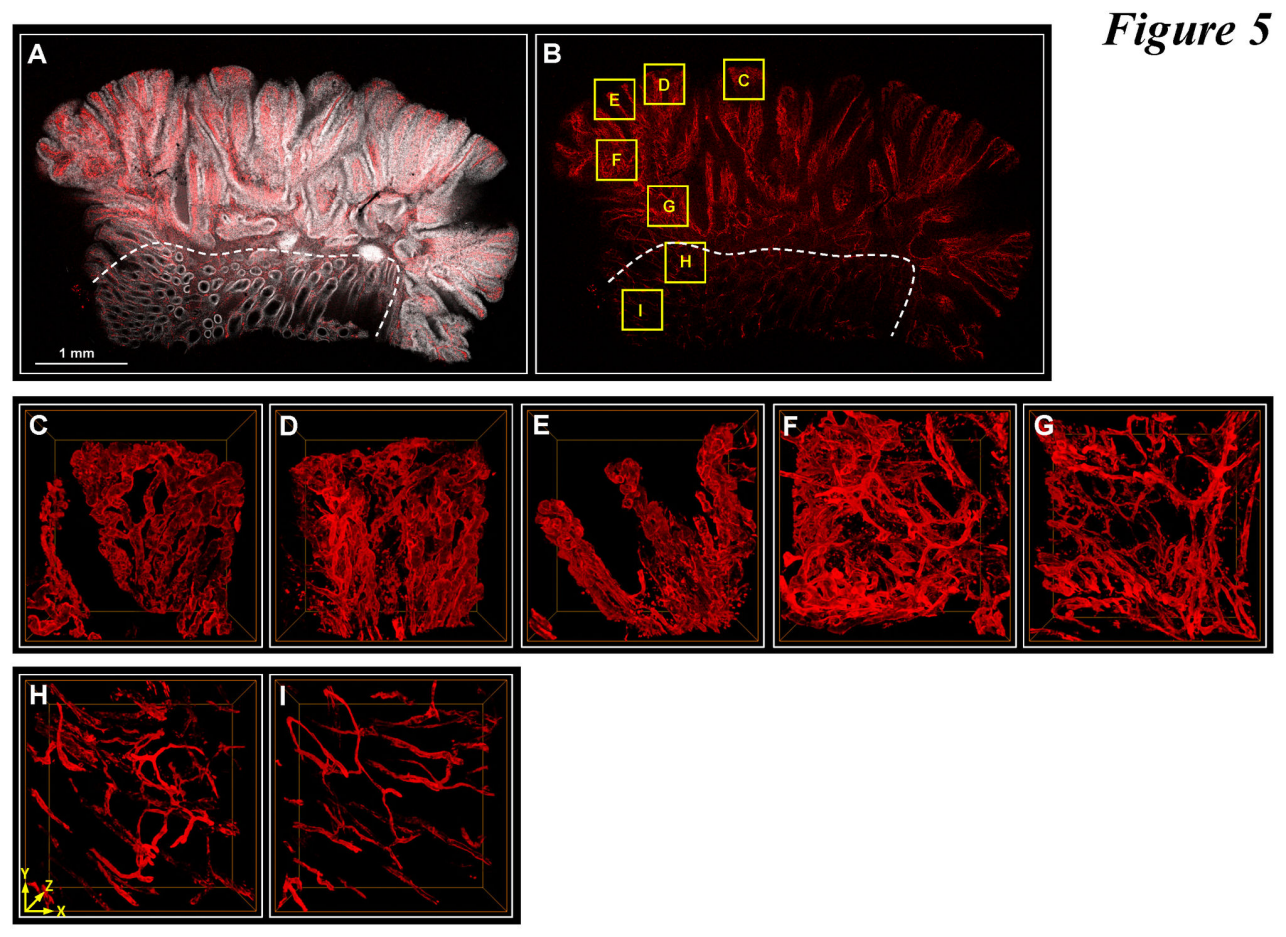
3-D imaging and illustration of microvessels in adenocarcinoma. (**A** and **B**) Tissue map of tumor specimen. Gray: nuclei. Red: CD34. Six images were stitched (derived from the tile scanning mode of confocal microscopy) to provide a gross view of the tissue structure across the adenocarcinoma and the apparent normal domain (the dotted line indicates the boundary). Seven regions of the tissue were marked for zoom-in investigation (boxes C-I). (**C**-**G**) 3-D projection of microvessels in adenocarcinoma. Panels C-G are the zoom-in examination of boxes C-G in panel B. In these regions, the microvessels are dilated and tortuous with extensive branches in comparison with the capillaries around the normal crypts (Figure 3F) and those in panels H and I. (**H** and **I**) 3-D projection of microvessels in the apparent normal region (controls, boxes H and I in panel B). Dimensions of each image stack: 460 (X) × 460 (Y) × 200 (Z, depth) μm.

Due to the irregular structure of the colorectal carcinoma, we first surveyed the specimen using tile scanning and image stitching algorithms to outline the colonic structure across the diseased and the (apparent) normal domains (Figures 5A and B). As can be seen, based on the nuclear signals, the adenocarcinoma spreads on the top and right sides of the gross view against the normal domain, which consists of intact crypt structures (below the line). In Figures 5C-I, we zoomed in and presented seven image stacks, five in the adenocarcinoma and two in the normal domain (boxes in Figure 5B).

The gross view and projections in Figure 5 reveal two features of the diseased tissue. First, the microvessels in the adenocarcinoma were dilated and tortuous with irregular branches and higher vascular density in comparison with those in the apparent normal region (comparison between Figures 5C-G and 5H-I). Second, the vascular abnormalities and increase in microvessel density were associated with the loss of the honeycomb-like pattern of crypts and increased nuclear signals in the tumor domain (Figure 5A), suggesting an ongoing process of mucosal remodeling and irregular mucosal proliferation likely aided and/or driven by angiogenesis in the tumor.

**Figure 6:**
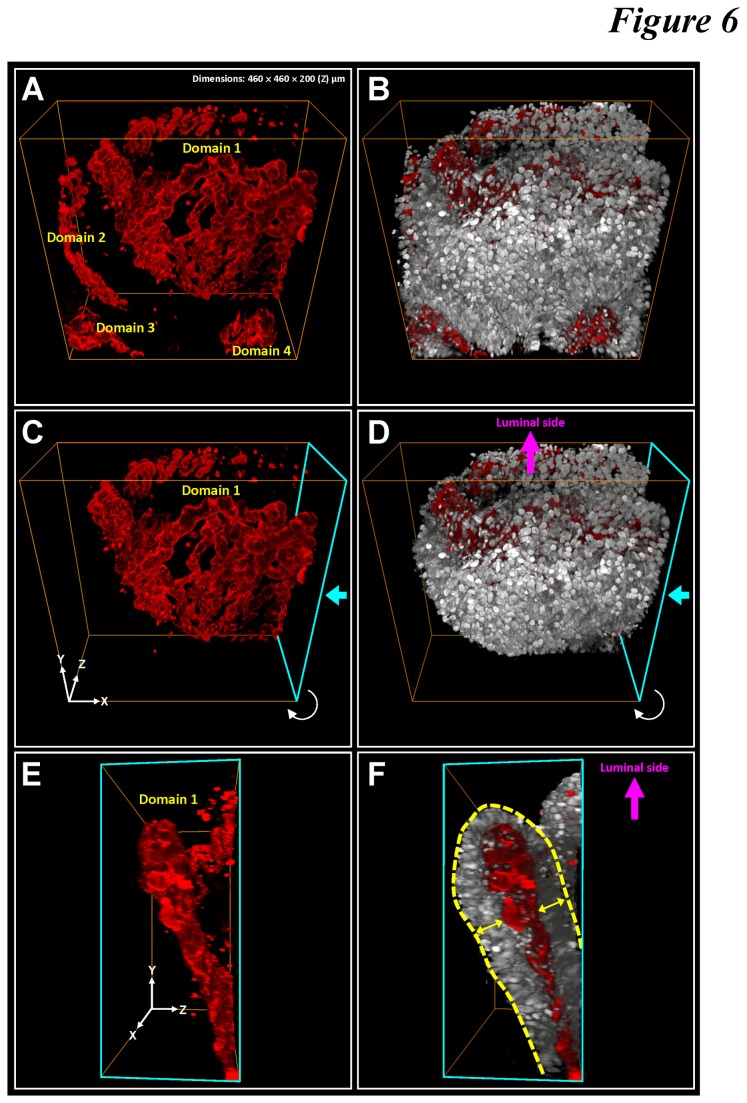
Layer-like microvascular network folded in space in adenocarcinoma. (**A** and **B**) 3-D projection of tumor microstructure (gray: nuclei) and vasculature (red: CD34). Four vascular domains were labeled in the image stack. Prominent nuclear signals were seen around the microvessels. (**C**-**F**) Layer-like vasculature folded in space and attached with perivascular cuffs of tumor cells. The signals were segmented from Domain 1 in panel A. Two viewing angles are presented here. The cyan arrows in panels C and D indicate the front side of the projections shown in panels E and F. The yellow arrows in panel F indicate the perivascular cuffs of tumor cells, forming a tumor cell-capillary-tumor cell sandwich structure folded in space (panels C and D). Panoramic projection of the image stack and an additional example of the layer-like microvascular network are presented in Video S4.

In addition, through in-depth projections from different viewing angles (Figure 6 and Video S4), we observed the structural pattern of the tissue outgrowth in adenocarcinoma. Microscopically, the adenocarcinoma carried folded sheets of vasculature (Figures 6A, C, and E) attached with perivascular cuffs of tumor cells forming a sandwich structure (Figures 6B, D, and F). 

Contrary to the normal mucosa with an array of honeycomb-like crypts (Figure 1C and the numerical numbers in Figure 4), the perivascular tumor cells of adenocarcinoma were arranged in layers (Figure 1D, Figure 6, and Video S4), providing the potential geometric structure to increase the endothelium-tumor cell contacts. Compared with the honeycomb-like structure, this arrangement allowed for higher carcinoma cell and vascular densities in space, creating a potential niche to facilitate tumor growth in the microenvironment.

### Quantitation of spatial microvessel density

We have demonstrated the remodeling of colonic microstructure and vasculature in adenocarcinoma. Using the digital image data, we next quantified the microvessel densities in the normal and diseased regions by dividing the voxels occupied by the microvessels with those of the tissue structures -- normal crypts (control) or carcinoma -- to estimate the angiogenesis in tumor biopsies. 

First, we examined the control microvessels around the normal crypts. Table 1 lists the spatial microvessel densities of the normal mucosa (>5 cm away from the tumor site) derived from two individuals, together with information about their gender, age, and location of the sampled colon segments. On average, 2.3% of the mucosal volume was occupied by the microvessels with no statistical difference between the biopsies acquired from the two individuals. 

We next compared the 3-D and 2-D quantitation of the microvessels in and around the adenocarcinoma using the seven regions sampled in Figure 5 (Subject 1 in Table 1). In 3-D quantitation, we observed a significant increase in the spatial microvessel density to 12.6% in the diseased area (regions C-G in Figure 5) relative to 3.2% in the mucosa adjacent to adenocarcinoma (regions H and I in Figure 5), or by 3.9-fold. The increase is by 6.0-fold relative to the microvessel density away from the tumor site (2.1%, Subject 1 in Table 1). 

In the 2-D microvessel analysis, we examined the same tumor regions to acquire the number of CD34-enclosed areas on the confocal micrographs at 15, 30, and 60 μm under the specimen surface. The last column of Table 2 lists the microvessel counts at 30 μm under the surface over a field area of 0.21 mm^2^. As can be seen, similar to the 3-D analysis, the 2-D microvessel counts also indicate a higher blood vessel content in the tumor regions (except region C) relative to the control. The major difference between the 3-D and 2-D data sets, however, is at the standard deviations, with the former (12% of the mean) substantially smaller than the latter (52%). Similarly, the standard deviations of the 2-D microvessel analysis at 15 μm (54% of mean) and 60 μm (25%) under the tissue surface (footnote of Table 2) also show higher variation in the 2-D analysis of vessel counts.

**Table 2:** Quantitation of microvessels in human biopsies of colorectal carcinoma.

		3-D analysis Spatial microvessel density (microvessel volume/tissue volume) × 100%	2-D analysis Microvessel counts**^*a*^** (microvessel number/per field area of 0.21 mm^2^)
Tumor (Regions in Figure 5)	C	12.1	14.0
	D	14.6	30.3
	E	10.6	29.0
	F	13.3	62.3
	G	11.9	53.3
	Average (C-G)	12.6 ± 1.5 (mean ± SD)**^*b*^**	38.2 ± 20.0 (mean ± SD)***^b^***,***^c^***
Adjacent to tumor (control)	H	3.8	21.3
	I	2.5	20.0
	Average (H & I)	3.2	20.7

***^a^*** Counts were evaluated by 3 independent examiners. ***^b^*** SD: standard deviation. ***^c^*** The analysis was taken at 30 μm under the tissue surface. The (mean ± SD) values at 15 and 60 μm under the tissue surface are (30.0 ± 16.1) and (26.0 ± 6.6), respectively.

 The remodeled vascular morphology in colorectal carcinoma likely contributed to the larger standard deviation in the 2-D tissue analysis. In Figure 6, we revealed that the microvessels were heterogeneously distributed in the tumor microenvironment with a folded layer-like structure. The viewing angle of the vascular structure therefore influenced the morphology and amount of the blood vessels presented on a 2-D micrograph, which is similar to the different faces of the vascular structure presented in Figure 6. In 3-D analysis, however, the microvessel density was measured and derived from a volume, which is orientation-free in quantitation, leading to a reduced standard deviation among the acquired data.

## Discussion

Microscopic analysis of the vasculature in colorectal carcinoma plays an important role in understanding tumor progression and malignancy. However, due to the geometry of the vascular network, standard 2-D histology is limited in providing information about the network architecture with a 3-D continuum. To facilitate 3-D microscopy, in this research we prepared transparent human colorectal biopsies for confocal imaging of the vascular network in space. The voxel-based image data were used for morphology reconstruction, projection, and digital analysis to characterize the tumor microstructure and vasculature in an integrated fashion. Our work advances the field of 3-D histology on establishing: (1) optical clearing of human colorectal tumor biopsies for deep-tissue microscopy, (2) 3-D qualitative and quantitative analyses of colorectal microvessels, and (3) the integration of 3-D microscopy with the standard 2-D immunohistochemistry based on the reversibility of the clearing process (Figure 4). These three new aspects/features of 3-D histology will help future investigators apply optical clearing to investigate the colorectal microstructure and vasculature in health and disease.

Although our optical approach demanded time for tissue clearing (overnight immersion) and image acquisition (one hour for a typical 200-μm image stack), the additional Z-axis information on the X/Y plane offered us an extra dimension to characterize the tumor microenvironment. The stereo projections of adenocarcinoma from various angles helped identify the spatial abnormalities, including the layer-like tumor vasculature with tortuous vessel walls to serve as the feeding bed for the perivascular tumor cells (Figure 6 and Video S4). Both the blood vessels and tumor cells were folded in space to increase the vascular and cellular densities, changing from the honeycomb-like pattern of normal crypts. Note that the normal mucosa is functionally structured to maximize absorption (Figure 3), yet the remodeled tumor microenvironment seeks to create a niche with increased cellular and vascular densities to locally enrich factors such as cytokines and nutrients to stimulate proliferation.

In addition to revealing the spatial information of microvessels, our imaging approach is also unique in that it offers two features to ensure the robustness of the staining/imaging outcomes to prepare for potential future clinical evaluation of tumor biopsies. First, in Figure 4 we demonstrate that the optically cleared mucosa can be retrieved after 3-D microscopy to perform the standard microtome-based histology, including H&E staining and immunohistochemistry. The integration will allow future investigators to have two sets of image data from the same specimen -- one as the standard 2-D micrographs and the other as an image stack for 3-D projection -- to specify the malignancy. Second, by overcoming the tissue opacity, we were able to increase the image depth of the colorectal carcinoma up to 200 μm (consisting of 81 2.5-μm optical sections) while maintaining the resolution power (of resolving the adjacent nuclei), alleviating the problem of incomplete sampling in analysis of the colorectal biopsies [14,38-40].

In Table 2, we compared the 3-D and 2-D quantitation of microvessels. In 3-D quantitation, we attributed the smaller standard deviation to volumetric vascular analysis rather than counting microvessels on the 2-D micrograph with a restricted viewing angle. Note that regarding the viewing angle of colorectal biopsies, prior studies have indicated the benefit of proper orientation of mucosal specimens on improving diagnostic accuracy [41]. Re-embedding the biopsy has been used, when necessary, to create well-oriented specimens for 2-D microscopy [14,42,43]. In 3-D microscopy, however, spatial features of tissue components can be digitally projected and analyzed in a panoramic fashion (Figure 6 and Videos S2-4), relieving the constraint on the viewing angles of colorectal biopsies.

By revealing the histological information in a space continuum, our optical and microscopic approach provides a comprehensive way of examining and presenting the structures of colorectal microvessels in health and carcinoma. However, the limitation of the technique needs to be addressed. Although the optically cleared colorectal biopsies allowed deep-tissue microscopy, the focal depth was limited within the 250-μm range, in which high-resolution microscopy was maintained with the resolving power to identify the adjacent nuclei and the diameter of capillaries. While it is desirable to extend the imaging depth to visualize the colorectal biopsy *in toto*, maintaining the resolution power is crucial for pathologists to define the malignancy, such as to identify the mucosal boundary to inspect whether the tumor lesion has invaded the submucosal domain (Figure 1A). This level of tissue information cannot be provided by the magnetic resonance imaging (MRI) and positron emission tomography (PET) scan, which focus on *in vivo* and macro-scale tissue information (millimeter and larger). 

In conclusion, we developed an optical approach to systematically integrate 3-D imaging, illustration, and quantitation of the vascular content in the human colorectal biopsies. Prior to this research, the intrinsic opacity of colorectal carcinoma has hindered in-depth observation of the tumor microstructure and vasculature. We show that the imaging hurdle can be overcome by optical clearing to facilitate visualization and quantitation of microvessels by 3-D histology with image projection, rendering, and segmentation. Future work will focus on applying this vascular imaging approach to other gastrointestinal tissues, including the esophagus, stomach, and pancreas, and to lymphatic vessels in malignancy to better understand and characterize the tumor microenvironment for diagnosis and therapy.

## Supporting Information

Figure S1 **Examination of the immunostaining variables: the source of antibody.** (**A**-**F**) In-depth projections of the mucosal vasculature derived from three sources of antibodies to label the blood vessels. Depth: 150 μm. Panels A and D: mouse anti-CD31 antibody (Thermo, Fremont, CA, USA, cat# MS-353-S0). Panels B and E: mouse anti-CD34 antibody (Bio SB, Santa Barbara, CA, USA, cat# BSB 5230). Panels C and F: rabbit anti-CD34 antibody (Epitomics, Burlingame, CA, USA, cat# 2150-1). The center parts of panels A-C were enlarged to reveal the noise signals in panel D (smear) and F (dots) derived from the mouse anti-CD31 and rabbit anti-CD34 staining, respectively. In this paper, we show the results of vascular staining with the antibody purchased from Bio SB (panels B and E). (**G**) Confirmation of the vascular morphology derived from the mouse anti-CD34 antibody. Individual and merged presentations of confocal (left) and transmitted light (middle) micrographs verify the locations of blood vessels underneath the colonic epithelium. Arrows indicate the locations of the vascular lumen. Red: CD34. Green: nuclei. Images were taken under the same view.
(PDF)

Figure S2 **Examination of the immunostaining variables: the kinetics of antibody diffusion in the specimen.** (**A**) Illustration of the experimental setup to test the diffusion kinetics of CD34 antibody (vender: Bio SB) in the specimen of human colonic mucosa. (**B**-**D**) CD34-labeled mucosal vasculature in the specimen after one, four, and 16 hours of primary antibody staining. The incubation time for the secondary antibody (Alexa Fluor 633-conjugated goat anti-mouse antibody, Invitrogen) staining was one day. Signal intensities are presented in grayscale, red (signal saturation), and blue (no signals). The signal profile analysis (lower panels, offered by the Zeiss Zen software) shows the signal intensity along the red line at the center of the micrograph. The signal peaks are marked with yellow arrows. Panel D indicates homogeneous CD34 staining across the mucosal layer after 16 hours of incubation.
(PDF)

Video S1 **Side-by-side comparison between the saline control and the optically cleared specimen in deep-tissue confocal microscopy.** Specimens: human colonic mucosa with CD34-labeled vasculature. The control specimen (left, in saline) shows a drastic decline of the fluorescence signals along the focal depth. In comparison, the optically cleared specimen maintains high signal contrast (right). Still images at depths of 60, 90, 120, and 150 μm are presented in Figure 2. Total depth: 150 μm. Increment: 2.5 μm.
(MP4)

Video S2 **In-depth microscopy of microvessels in optically cleared mucosa.** The video shows the crypt and the surrounding vasculature in continuous optical sections and in 360-degree projection. Gray: nuclei. Red: CD34-labeled blood vessels. Dimensions of the scanned volvolume: 460 μm (X) × 460 μm (Y) × 200 μm (Z, depth).
(MP4)

Video S3 **360-degree projection of microvessels in wholemount specimen.** The capillaries form a honeycomb-like pattern beneath the crypt openings. In the projection, a 2-D micrograph was added to illustrate the association between the microvessels and crypts. Dimensions of the scanned volvolume: 460 μm (X) × 460 μm (Y) × 200 μm (Z, depth).
(MP4)

Video S4 **360-degree projection of the layer-like vascular network in adenocarcinoma.** Two examples are presented in this video. In the second example, the nuclear signals (gray) were digitally removed in rotation to reveal the sandwiched microvessels. Dimensions of the scanned volvolume: 460 μm (X) × 460 μm (Y) × 200 μm (Z, depth) for both image stacks.
(MP4)
