# Correction: 3-D Visualization and Quantitation of Microvessels in Transparent Human Colorectal Carcinoma

**DOI:** 10.1371/annotation/b9f7e31f-460e-4fef-bfdf-038fadbf4c1b

**Published:** 2013-12-17

**Authors:** Yuan-An Liu, Shien-Tung Pan, Yung-Chi Hou, Ming-Yin Shen, Shih-Jung Peng, Shiue-Cheng Tang, Yuan-Chiang Chung

This article was republished to correct a typographical error in the title.

