# Supplementary figures and images for: 3-D Visualization and Quantitation of Microvessels in Transparent Human Colorectal Carcinoma
Source: PLoS One. 2013 Nov 29;8(11):e81857. doi: 10.1371/journal.pone.0081857 (PMC3843693; doi:10.1371/journal.pone.0081857)

## *Supplemental Figure S1*

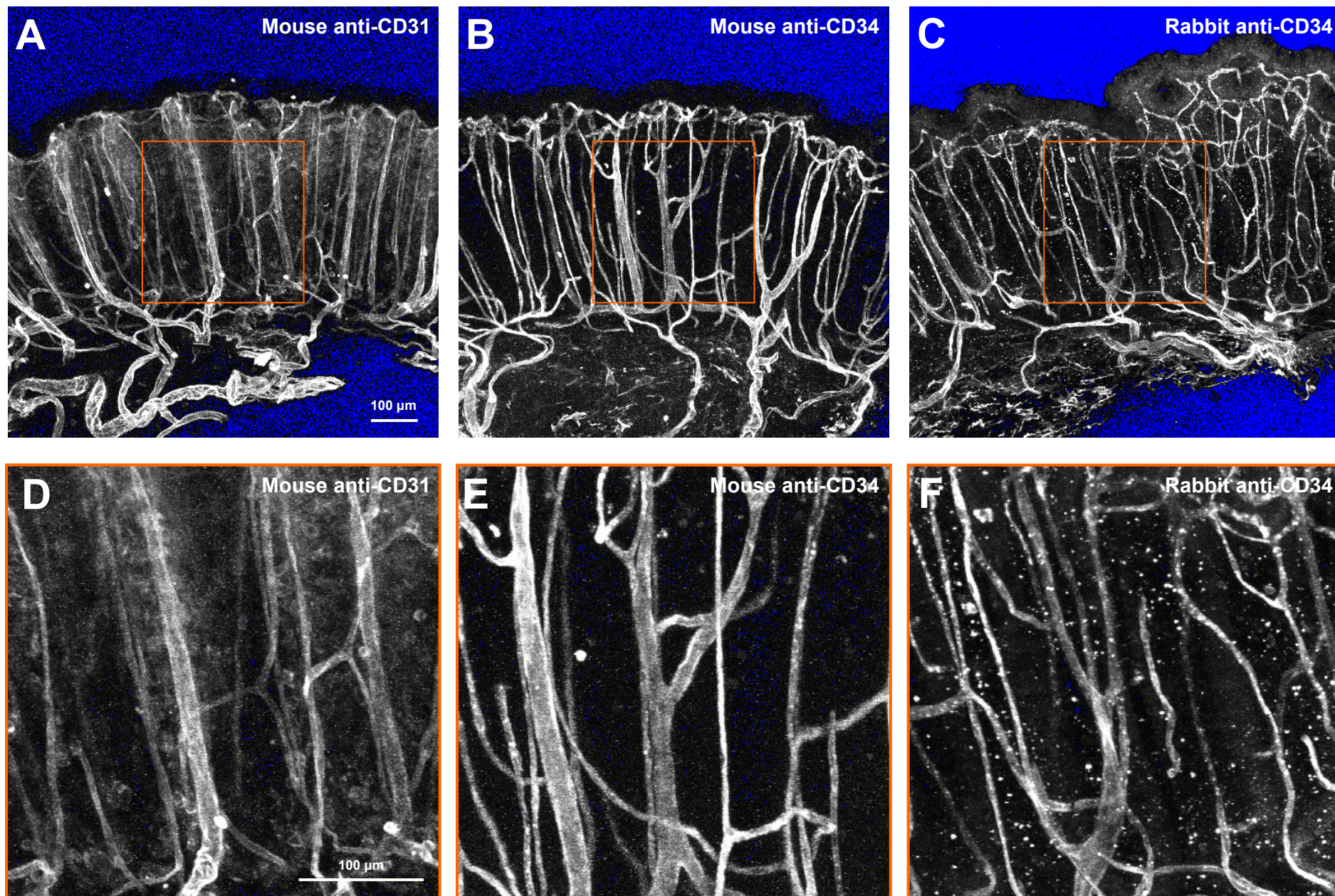

## *Supplemental Figure S1, continued*

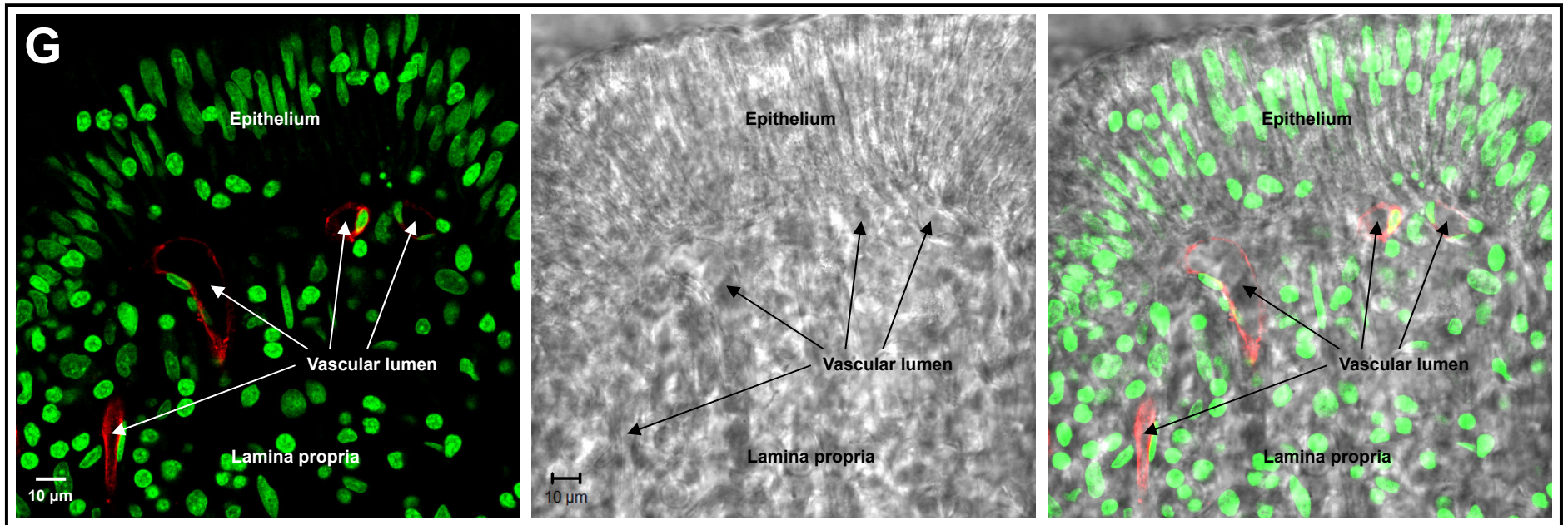

Supplement: Figure S1 — Examination of the immunostaining variables: the source of antibody. (A-F) In-depth projections of the mucosal vasculature derived from three sources of antibodies to label the blood vessels. Depth: 150 μm. Panels A and D: mouse anti-CD31 antibody (Thermo, Fremont, CA, USA, cat# MS-353-S0). Panels B and E: mouse anti-CD34 antibody (Bio SB, Santa Barbara, CA, USA, cat# BSB 5230). Panels C and F: rabbit anti-CD34 antibody (Epitomics, Burlingame, CA, USA, cat# 2150-1). The center parts of panels A-C were enlarged to reveal the noise signals in panel D (smear) and F (dots) derived from the mouse anti-CD31 and rabbit anti-CD34 staining, respectively. In this paper, we show the results of vascular staining with the antibody purchased from Bio SB (panels B and E). (G) Confirmation of the vascular morphology derived from the mouse anti-CD34 antibody. Individual and merged presentations of confocal (left) and transmitted light (middle) micrographs verify the locations of blood vessels underneath the colonic epithelium. Arrows indicate the locations of the vascular lumen. Red: CD34. Green: nuclei. Images were taken under the same view. (PDF) [file pone.0081857.s001.pdf]

## Supplemental Figure S2

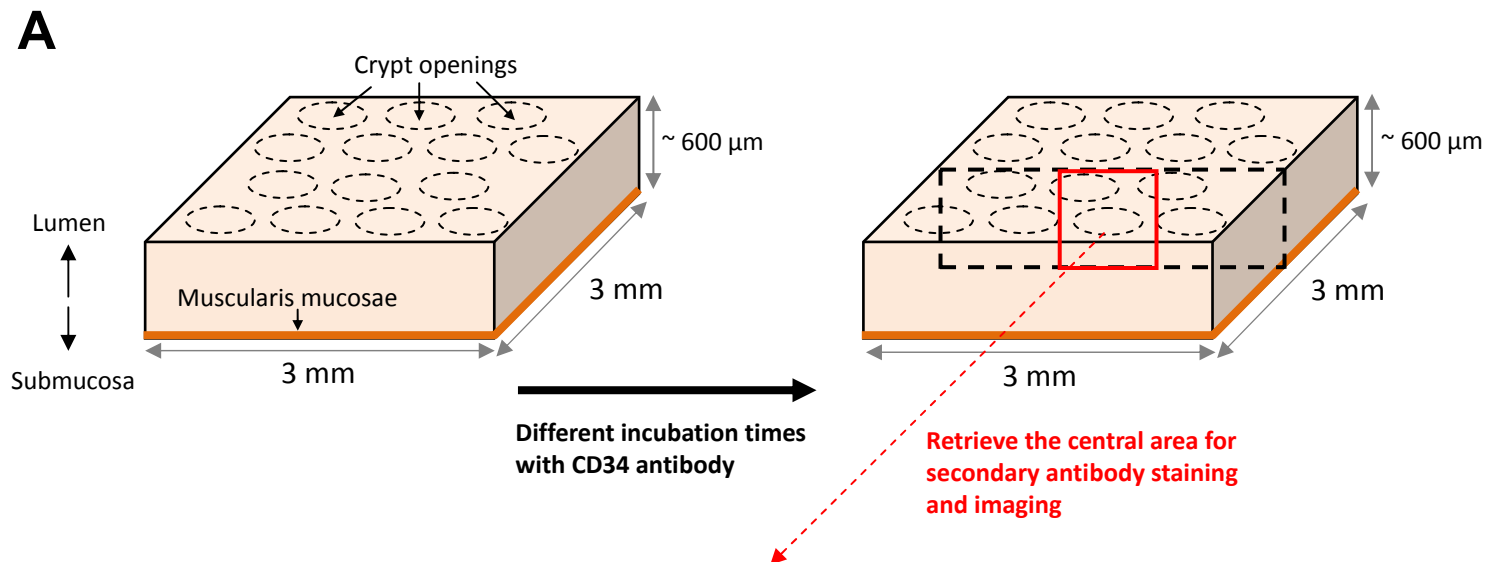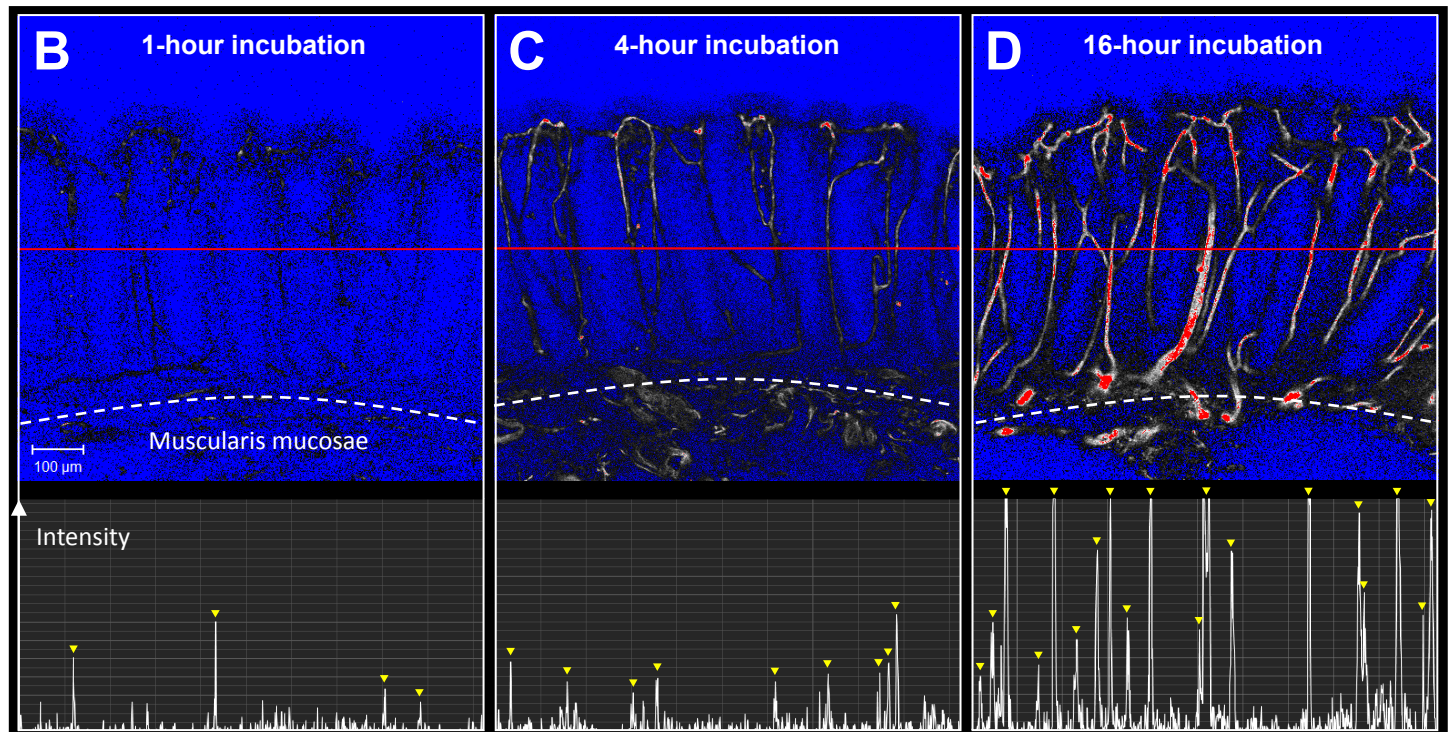

Supplement: Figure S2 — Examination of the immunostaining variables: the kinetics of antibody diffusion in the specimen. (A) Illustration of the experimental setup to test the diffusion kinetics of CD34 antibody (vender: Bio SB) in the specimen of human colonic mucosa. (B-D) CD34-labeled mucosal vasculature in the specimen after one, four, and 16 hours of primary antibody staining. The incubation time for the secondary antibody (Alexa Fluor 633-conjugated goat anti-mouse antibody, Invitrogen) staining was one day. Signal intensities are presented in grayscale, red (signal saturation), and blue (no signals). The signal profile analysis (lower panels, offered by the Zeiss Zen software) shows the signal intensity along the red line at the center of the micrograph. The signal peaks are marked with yellow arrows. Panel D indicates homogeneous CD34 staining across the mucosal layer after 16 hours of incubation. (PDF) [file pone.0081857.s002.pdf]
